# Metabolic Effects of CX3CR1 Deficiency in Diet-Induced Obese Mice

**DOI:** 10.1371/journal.pone.0138317

**Published:** 2015-09-22

**Authors:** Rachana Shah, Sean M. O’Neill, Christine Hinkle, Jennifer Caughey, Stephen Stephan, Emma Lynch, Kate Bermingham, Gina Lynch, Rexford S. Ahima, Muredach P. Reilly

**Affiliations:** 1 Division of Endocrinology and Diabetes, The Children’s Hospital of Philadelphia, Philadelphia, Pennsylvania, United States of America; 2 Cardiovascular Institute, Perelman School of Medicine at the University of Pennsylvania, Philadelphia, Pennsylvania, United States of America; 3 Division of Endocrinology, Diabetes and Metabolism, Perelman School of Medicine at the University of Pennsylvania, Philadelphia, Pennsylvania, United States of America; Hospital Infantil Universitario Niño Jesús, CIBEROBN, SPAIN

## Abstract

The fractalkine (CX3CL1-CX3CR1) chemokine system is associated with obesity-related inflammation and type 2 diabetes, but data on effects of Cx3cr1 deficiency on metabolic pathways is contradictory. We examined male C57BL/6 Cx3cr1^-/-^ mice on chow and high-fat diet to determine the metabolic effects of Cx3cr1 deficiency. We found no difference in body weight and fat content or feeding and energy expenditure between Cx3cr1^-/-^ and WT mice. Cx3cr1^-/-^ mice had reduced glucose intolerance assessed by intraperitoneal glucose tolerance tests at chow and high-fat fed states, though there was no difference in glucose-stimulated insulin values. Cx3cr1^-/-^ mice also had improved insulin sensitivity at hyperinsulinemic-euglycemic clamp, with higher glucose infusion rate, rate of disposal, and hepatic glucose production suppression compared to WT mice. Enhanced insulin signaling in response to acute intravenous insulin injection was demonstrated in Cx3cr1^-/-^ by increased liver protein levels of phosphorylated AKT and GSK3**β** proteins. There were no differences in adipose tissue macrophage populations, circulating inflammatory monocytes, adipokines, lipids, or inflammatory markers. In conclusion, we demonstrate a moderate and reproducible protective effect of Cx3cr1 deficiency on glucose intolerance and insulin resistance.

## Introduction

Inflammation plays a central role in the pathophysiology of obesity-related cardiometabolic disease. Fractalkine (CX3CL1), a chemokine implicated in chronic inflammatory disease[1–5] and its receptor (CX3CR1) have long been considered potential targets in atherogenic cardiovascular disease (CVD)[6, 7]. CX3CL1 is expressed widely, and undergoes marked induction by inflammatory cytokines[2, 3, 8–10]. As a chemokine, CX3CL1 promotes leukocyte adhesion and migration to vascular lesions in atherosclerosis models[11]. Binding to its receptor triggers signaling via phosphatidylinositol 3-kinase (PI3K)/Akt and MAPK promoting leukocyte activation and survival[8, 9, 12, 13].

The potential role of CX3CL1 in the pathogenesis of diabetes mellitus has emerged more recently[7, 14]. Our group found that CX3CL1 is an adipocyte-derived inflammatory chemokine that promotes monocyte adhesion to adipocytes, has increased mRNA and protein levels in obese (compared to lean) adipose tissue, and that plasma levels are significantly elevated in diabetic subjects compared to non-diabetic controls[7]. Polymorphisms in the CX3CR1 gene have also been associated with obesity and metabolic traits[14, 15] [7]. A more recent study suggests that Cx3cr1^-/-^ mice might have a defect in beta cell insulin secretion[16], suggesting a novel pancreatic mechanism by which the CX3CL1 system affects glucose metabolism. Given these associations, strongly suggesting involvement of the CX3CL1-CX3CR1 pathway in diabetes and metabolic traits, we aimed to perform comprehensive metabolic phenotyping to determine whether abolition of this pathway ameliorated obesity-induced metabolic derangements in a mouse model.

In these studies, we examined the metabolic effects of Cx3cr1 deficiency in mice. We compared body composition, energy expenditure, inflammatory markers and metabolic phenotypes of Cx3cr1^-/-^ to their wild-type littermates on a high-fat diet. After finding modest but reproducible attenuation of diet-induced glucose intolerance, we assessed insulin sensitivity using hyperinsulinemic-euglyemic clamp and tracer kinetics. We found no difference in body weight and fat content or feeding and energy expenditure between Cx3cr1^-/-^ and WT. Cx3cr1 deficiency improved insulin sensitivity at hyperinsulinemic-euglycemic clamp, and also enhanced insulin signaling in response to acute intravenous insulin injection. In contrast, Cx3cr1^-/-^ did not alter pancreatic insulin secretion.

## Methods

### Mice and diets

C57BL/6 Cx3cr1^-/+^ heterozygous mice were received from the Charro laboratory[17] and crossed to generate littermate knockouts (Cx3cr1^-/-^) and wild type (WT). Male mice were used in the experiments described. Beginning at 9–10 weeks of age, the mice were fed a 45% kcal high-fat diet (D12451, Research Diets) for 4–24 week periods. Four cohorts of mice were studied: 4 week HFD, 12 week HFD, 18 week HFD and 24 week HFD. Each group consisted of 15–30 WT and 15–30 Cx3cr1^-/-^ mice; we included all males with each genotype born within a 5-day period. Mice were housed in a specific pathogen-free facility on a 12 hr light/12 hr dark cycle and given free access to food and water. WT and Cx3cr1^-/-^ mice were housed together, 4–5 mice per cage, to prevent cage-specific effects from confounding the data. For any experiments performed on a subset of mice, mice were chosen at random by drawing numbers. All animal use was in compliance with the Institute of Laboratory Animal Research Guide for the Care and Use of Laboratory Animals and approved by the Institutional Use and Care of Animals Committee (IACUC) at the University of Pennsylvania (protocol number: 803275). Details of mouse experiment are summarized in **S1 Checklist.**

### Energy homeostasis

The mice were weighed weekly. Body composition was determined with ^1^H magnetic resonance spectroscopy (Echo Medical Systems, Houston, TX). Feeding, energy expenditure and locomotor activity were measured using a Comprehensive Laboratory Animal Monitoring System (CLAMS, Columbus Instruments) as previously described[19].

### Glucose homeostasis

Glucose tolerance test (GTT) was performed at 8 AM after overnight (16 hours) fasting. After measuring tail blood glucose with a glucometer, 20% glucose solution (2 g/kg) was administered by intraperitoneal (i.p.) injection and tail blood glucose was measured at 0, 15, 30, 45, 60, 90, and 120 minutes by glucometer (AlphaTRAK, Abbott Laboratories, Alameda, CA). To assess insulin secretion, mice were fasted for 16 hours, tail blood glucose was measured with a glucometer and blood was collected, 20% glucose (1 g/kg) IP was administered and blood samples were collected at 5 and 15 minutes. Serum insulin was measured using ultra-sensitive mouse insulin ELISA (Crystal Chem Inc, Downers Grove, IL).

### Hyperinsulinemic euglycemic clamp

Studies with 3H and 14C tracers were performed at the Mouse Phenotyping, Physiology and Metabolism Core at the University of Pennsylvania School of Medicine as previously described[20]. An indwelling catheter was inserted into the right internal jugular vein under sodium pentobarbital anesthesia and extended to the right atrium. Four days after recovery of pre-surgery weight, the mice were fasted for 6 hours (7am-1pm), tail blood glucose was measured and a bolus injection of 5 μCi of [3–^3^H] glucose was administered intravenously followed by continuous intravenous infusion at 0.05 μCi min^-1^. Baseline glucose kinetics was measured for 120 min followed by hyperinsulinemic clamp for 120min. A priming dose of regular insulin (16mUkg−1, Humulin; Eli Lilly, Indianapolis, IN) was given intravenously, followed by continuous infusion at 2.5 mUkg−1min−1. A variable intravenous infusion of 20% glucose was infused to attain blood glucose levels of 120–140mg/dL. At the end of the clamp, 10 μCi 2-deoxy-D-[1-14C] glucose was injected to measure glucose uptake. The mice were euthanized, and liver, perigonadal adipose tissue and gastrocnemius muscle samples were excised, frozen in liquid nitrogen and stored at −80°C for analysis of glucose uptake. Rates of whole body glucose uptake and basal glucose turnover were measured as the ratio of the [3H] glucose infusion rate (d.p.m.) to the specific activity of plasma glucose. Hepatic glucose production (HGP) during clamp was measured by subtracting the glucose infusion rate (GIR) from the whole body glucose uptake (Rd). Under basal (fasting) conditions, the rate of Rd = HGP.

### Liver responses to acute insulin administration

To assess *in vivo* insulin signaling, the mice were fasted for 16 hours and either phosphate buffered saline or insulin at 2 mU g^-1^ body weight was injected via inferior vena cava. The mice were sacrificed after 5 minutes, and samples of liver and adipose were rapidly excised, freeze clamped in liquid nitrogen and then stored at -80C. Tissues were extracted with RIPA buffer (150 mM NaCl, 50 mM Tris, pH 7.6, 1% Triton X–100, 0.5% sodium deoxycholate, 0.1% SDS) supplemented with protease and phosphatase inhibitors), and immunoblotting was performed as described previously[21] using the following antibodies: Phospho-GSK-3β Phospho-AKT (Ser473), BetaActin (all from Cell Signaling Technologies, Denvers, MA).

### RNA extraction and real-time PCR analysis

Livers were isolated, frozen in liquid nitrogen, and stored at -80°C. Total RNA was isolated from tissues using Trizol (Invitrogen, Carlsbad, CA) and cDNA was generated from 0.5–1.0 mg total RNA using High Capacity cDNA Reverse Transcription Kits (Applied Biosystems). Real-time PCR analysis was performed using TaqMan Master Mix (Applied Biosystems) and the ABI7900 System (Applied Biosystems). Beta actin expression was used as the internal control for data normalization. Samples were assayed in duplicate and relative expression was determined using the 2^-ΔΔ^CT method.

### Blood and adipose flow cytometry

For blood, mice were anesthesized and blood (200 ul) was collected via retro-orbital puncture in heparinized micro-hematocrit capillary tubes (Fisher Scientific, Pittsburgh, PA). Red blood cells (RBCs) were lysed in 2 ml Pharm Lyse (BD Biosciences, San Jose, CA) for 15 min. Lysates were centrifuged and the pelleted blood leukocytes were resuspended in PBS/0.5%BSA before staining with the following fluorochrome-conjugated antibodies: CD45-FITC, Ter119-PE, CD115-APC, Ly6G-PE-Cy7, Ly6C-AF700, CD19-PerCP-Cy5.5, and CD3-APC-eF780 (all from eBioscience, San Diego, CA). Samples were incubated in antibody for 30 minutes, washed, stained with DAPI and analyzed on BD LSRII flow cytometer.

For adipose, the stromal vascular fraction (SVF) was isolated and prepared for flow cytometry according to the protocol published by Cho et al.[22], with minor modifications. Briefly, inguinal adipose samples were manually minced, digested immediately with Type I collagenase (Sigma Aldrich, St Luis, MO) for 30 min, and centrifuged to separate the SVF from mature adipocytes. SVCs were washed in 2 mL FACS buffer (PBS/1 mM EDTA/25 mM HEPES/ 1% heat-inactivated fetal bovine serum), centrifuged, and resuspended in 200 μL FACS buffer. After blocking with Fc block (BD Bioscience, San Jose, CA), SVF cells were stained with the following fluorochrome-conjugated antibodies: CD45-FITC, Ter119-PE, F4/80-PE-Cy7, CD11c-APC-eF780, and CD11b-AF700 (all from eBioscience) and CD301(MGL-1)-AF647 (AbD Serotec, Raleigh, NC). Samples were incubated in antibody for 30 minutes, washed, stained with DAPI and run on BD LSRII flow cytometer.

### Plasma biomarkers

Blood was collected via retro-orbital puncture in anesthesized mice. Adipokine markers were measured in duplicate (intra-assay CV = 5.2% for adiponectin, 14.3% for leptin, 7.1% for resistin) via Luminex multiplex assay. Plasma inflammatory markers (fractalkine, CCL2, and IL6) were measured by ELISA (R&D systems). Free fatty acids and lipid measurements were performed on the Cobas Mira-Plus biochemistry autoanalyzer (Hoffman la Roche; Basel, Switzerland).

### Sample Size and Statistics

Sample size calculations were performed to find the minimum number of mice per group needed to detect differences in OGTT glucose area under the curve (AUC) with 80% power. Based on results from a pilot study of WT mice, the calculated minimal sample size was 15. The data were analyzed using GraphPad Prism 5. Groups were compared using student’s T-test or Mann Whitney U for non-parametric variables. Data over time were analyzed using 2-way-Anova with post-hoc analysis. Data are expressed as means +/- standard deviations. Reported “n” refers to individual mice in each group.

## Results

### Cx3cr1^-/-^ does not affect weight, body composition, or energy homeostasis

**Fig 1:**
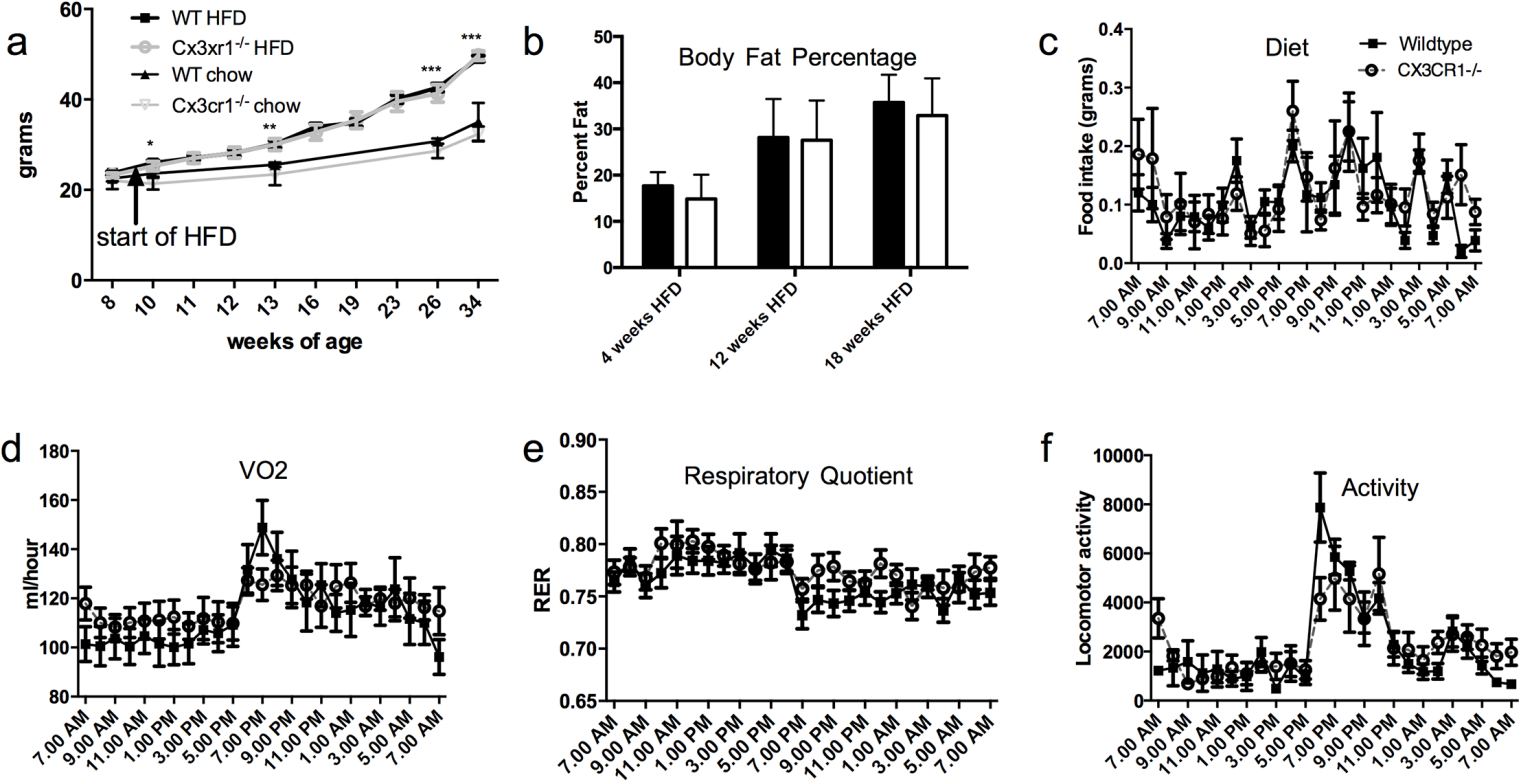
Cx3cr1^-/-^ has no effect on weight, body composition, or energy homeostasis. (a) Weight on chow diet (at 8 weeks) and weight gain over 24 weeks of feeding with 45% fat diet is identical between groups (n = 11 WT, 10 Cx3cr1^-/-^). The high-fat fed mice do show increased weight compared to age-matched chow-fed mice of each genotype (n = 6 WT, 8 Cx3cr1^-/-^) p = 0.02 at 2 weeks, 0.006 at 13 weeks, and <0.001 at 18 and 24 weeks HFD (b) At 4, 12, and 18 weeks body fat percentage via NMR was no different between groups (n = 15 WT/13 Cx3cr1^-/-^ at 4 weeks, 28 WT/30 Cx3cr1^-/-^ at 12 weeks, and 19 WT/14 Cx3cr1^-/-^ at 18 weeks HFD). Mice were identical in CLAMS metabolic measures of c) diet, d) VO2, e) respiratory quotient (RER) and f) activity (n = 10 WT, 8 Cx3cr1^-/-^) at 18 weeks HFD. Data are mean +/- SD. *p<0.05 **p<0.01 ***p<0.001 between groups.

WT and Cx3cr1^-/-^ mice had similar weights on both regular chow (23.3 +/- 1.55 grams vs. 23.7 +/- 1.35 grams at 8 weeks of age; p = NS) and high-fat diet (HFD) (49.0 +/- 2.23 grams vs. 49.7 +/- 3.25 grams after 24 weeks of diet).) (**Fig 1A**; p = NS between WT and Cx3cr1^-/-^ at each time point; n = 11 WT and 10 Cx3cr1^-/-^ shown, representative of 5 separate experiments). Both sets of high-fat fed mice were, however, heavier than chow fed WT and Cx3cr1^-/-^ mice (**Fig 1A**; 35.1 +/- 8.4 and 32.4 +/- 1.64 grams after 24 weeks. p = 0.02 at 2 weeks, 0.006 at 13 weeks, and <0.001 at 18 and 24 weeks HFD). Body composition also showed no differences between groups in body fat percentage with high-fat feeding for 4 weeks (17.7 +/- 2.93 vs. 14.86 +/- 5.24, n = 15 WT and 13 Cx3cr1^-/-^), 12 weeks (28.15 +/- 8.34 vs. 27.54 +/- 8.61, n = 28 WT, 30 Cx3cr1^-/-^), or 18 weeks (35.73 +/- 6.02 vs. 32.92 +/- 8.02, n = 19 WT and 14 Cx3cr1^-/-^) (**Fig 1B**; p = NS between WT and Cx3cr1^-/-^ at each time point). Feeding, energy expenditure, respiratory exchange ratio (RER) measured with CLAMS at 20 weeks high-fat diet were not altered by Cx3cr1 deficiency (**Fig 1C–1F;** p = NS by ANOVA for all measures, n = 10 WT and 8 Cx3cr1^-/-^).

### Cx3cr1^-/-^ reduces high-fat diet induced glucose intolerance without affecting insulin secretion

**Fig 2:**
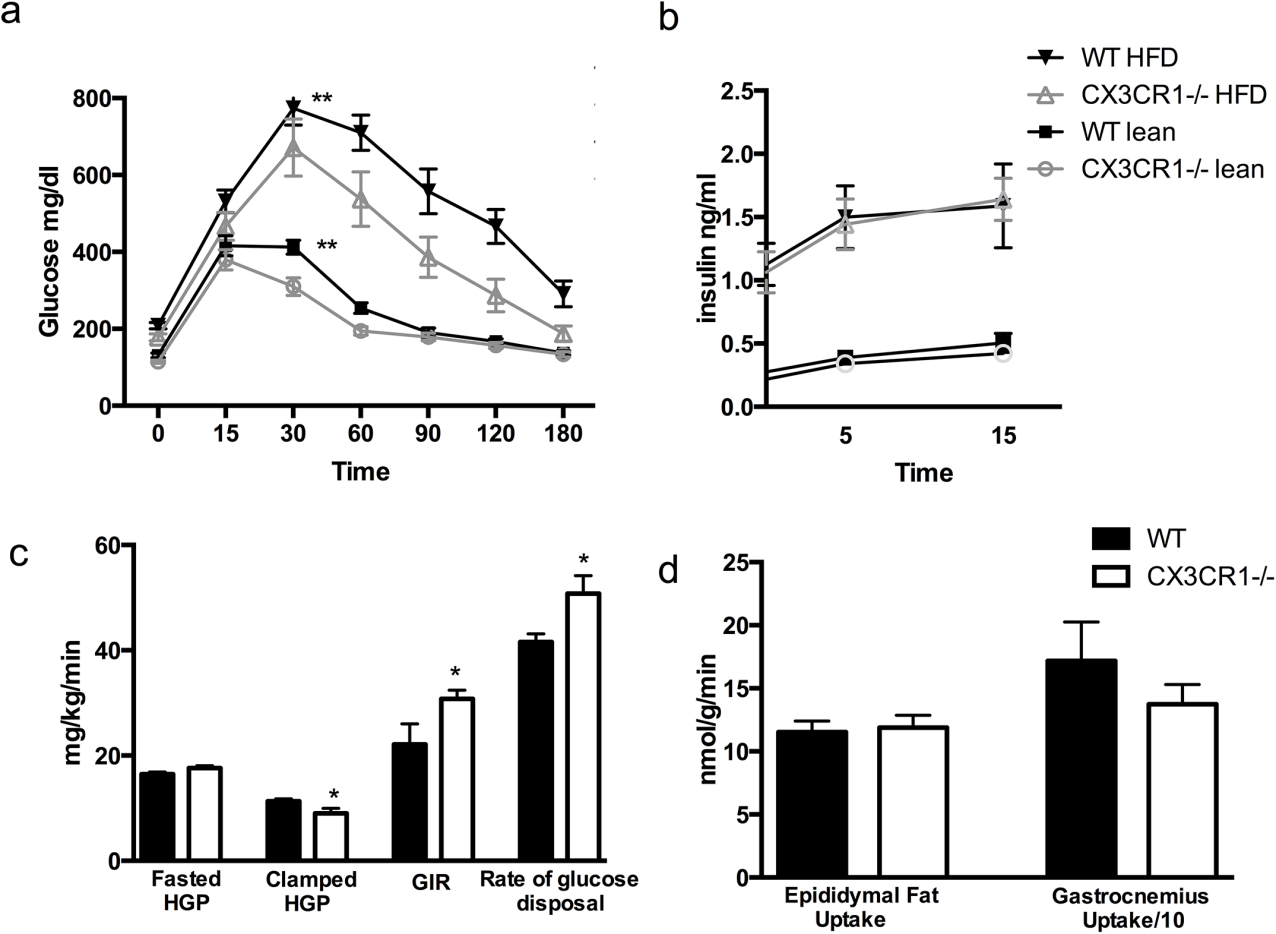
Cx3cr1^-/-^ reduces high-fat diet-induced glucose intolerance and improves insulin sensitivity at hyperinsulinemic euglycemic clamp without affecting insulin secretion. (a) Reduction in glucose excursions following intraperitoneal glucose tolerance test (n = 11 WT, 10 Cx3cr1^-/-^). Cx3cr1^-/-^ mice had reduced glucose area under the curve (AUC) at chow (1344 +/- 176 vs. 1590 +/- 127; p = 0.002) and 12 week HFD (2531 +/- 813 vs. 3182 +/- 435; p = 0.001). (b) Glucose-stimulated serum insulin values are not different between groups on chow or 12 week HFD (n = 16/group; p = 0.2 by ANOVA). (c) During hyperinsulinic euglyemic clamp on 24 week HFD (n = 10 WT and 9 Cx3cr1^-/-^), fasted hepatic glucose production (HGP) was higher, though not significantly different, in Cx3cr1^-/-^ mice (17.6 +/- 1.3 vs. 16.5 +/- 1.3 mg/kg/min; p = 0.07). HGP with insulin infusion during the clamp was reduced in Cx3cr1^-/-^ mice (9 +/- 2.97 vs. 11.3 +/- 1.57 mg/kg/min; p = 0.046). Glucose infusion rate required to maintain euglycemia was higher in Cx3cr1^-/-^ mice (41.8 +/- 13.1 vs. 30.3 +/- 5.6 mg/kg/min; p = 0.02). Rate of glucose disposal was higher in the Cx3cr1^-/-^ mice (50.8 +/- 10.2 vs. 41.6 +/- 4.87 mg/kg/min; p = 0.01). (d) There was no difference between groups in e) epididymal fat or f) gastrocnemius muscle uptake. *p<0.05 **p<0.01 between groups.

While both groups demonstrated glucose intolerance with HFD, the effect was lower in Cx3cr1^-/-^ mice suggesting reduction of HFD-induced glucose intolerance **(Fig 2A)**. Data shown are from one representative experiment (n = 11 WT and 10 Cx3cr1^-/-^); these findings were reproduced in 3 experiments. Cx3cr1^-/-^ mice had reduced glucose area under the curve (AUC) at chow (1344 +/- 176 vs. 1590 +/- 127; p = 0.002) and 12 week HFD (2531 +/- 813 vs. 3182 +/- 435; p = 0.001). In contrast, baseline and glucose-stimulated insulin levels (at 5 and 15 minutes) were not different between WT and Cx3cr1^-/-^ mice after 12-weeks HFD (p = 0.2 between groups by ANOVA, n = 8/group) **(Fig 2B)**.

The effects of Cx3cr1 deficiency on glucose fluxes during a hyperinsulinic-eugylemic clamp assessment in Cx3cr1^-/-^ and WT fed HFD for 24 weeks (n = 10 WT and 9 Cx3cr1^-/-^) are shown in **Fig 2C**. Fasting hepatic glucose production (HGP) trended higher in Cx3cr1^-/-^ mice compared to WT (17.6 +/- 1.3 vs. 16.5 +/- 1.3 mg/kg/min; p = 0.07), while the HGP during the clamp was lower in Cx3cr1^-/-^ vs. WT mice (9 +/- 2.97 vs. 11.3 +/- 1.57 mg/kg/min; p = 0.046). Thus, Cx3cr1^-/-^ mice had increased % HGP suppression compared to WT (48.4 +/- 18.4 vs. 30.7 +/- 12.5; p = 0.04; data not shown).The glucose infusion rate (GIR) required to maintain euglycemia during the clamp was higher in Cx3cr1^-/-^ mice (41.8 +/- 13.1 vs. 30.3 +/- 5.6 mg/kg/min; p = 0.02) as was the rate of glucose disposal (Rd) (50.8 +/- 10.2 vs. 41.6 +/- 4.87 mg/kg/min; p = 0.01), indicating an increase in peripheral insulin sensitivity in Cx3cr1^-/-^ on HFD. However, there were no differences in glucose uptake in perigonadal adipose or gastrocnemius muscle between WT and Cx3cr1^-/-^ mice **(Fig 2D)**. The increase in Rd in CX3CR1 deficiency may be due to enhanced insulin sensitivity in either adipose and muscle tissues, or other peripheral organs that were not analyzed in the current study.

### Cx3cr1^-/-^ enhances hepatic insulin signaling

**Fig 3:**
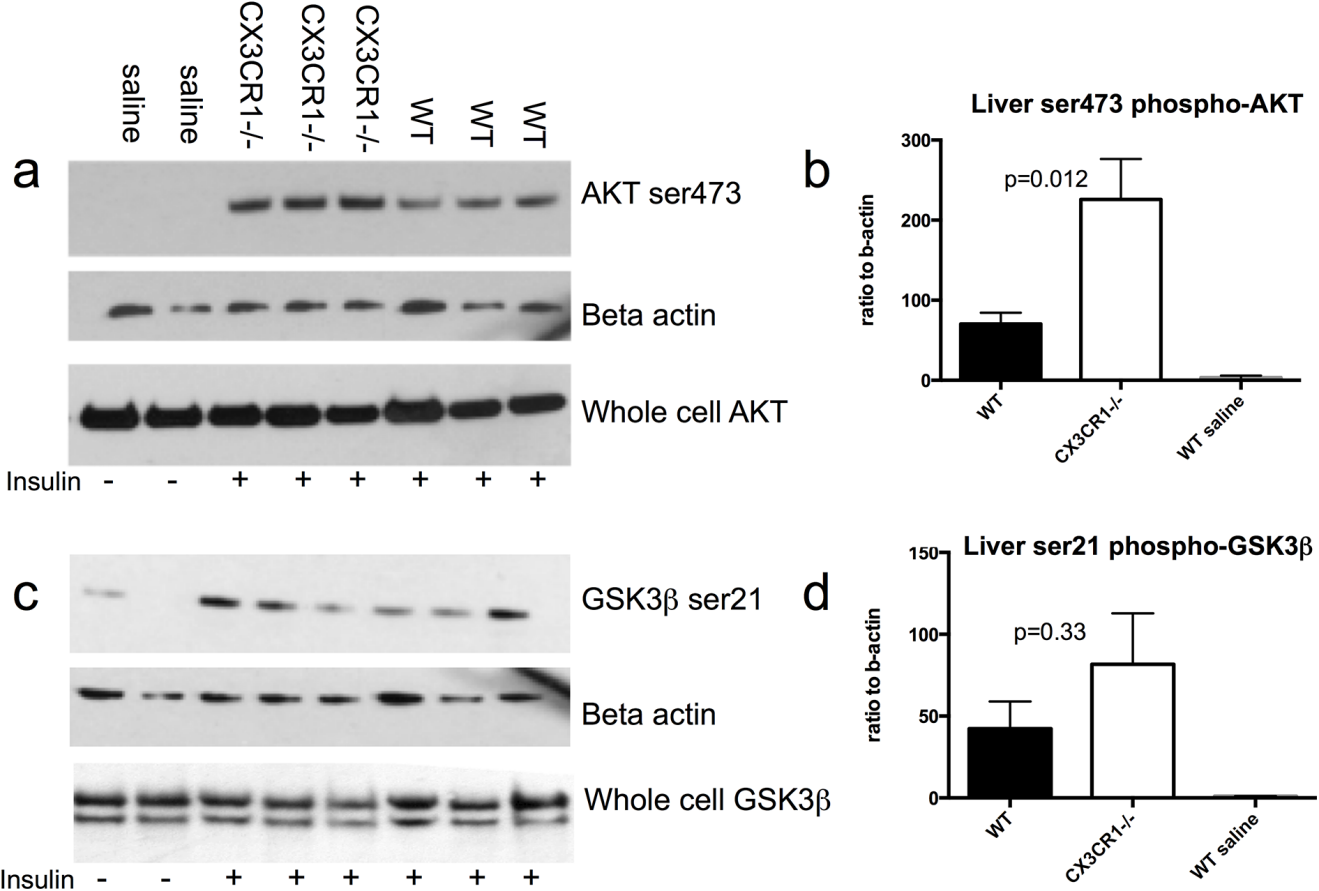
Cx3cr1^-/-^ enhances insulin signaling genes phosphorylated-AKT and phosphorylated-Glycogen synthase kinase 3 beta (GSK3β) in liver with high-fat feeding. After acute insulin stimulation, Cx3cr1^-/-^ mice had higher liver protein levels than WT mice by immunoblot of the insulin signaling genes (a-b) phosphorylated AKT (n = 3/group) and (c-d) serine 21-phosphorylated GSK3β (n = 3/group) after a 20-week high fat diet. Whole cell AKT and GSK3β were no different by group. WT saline-injected mice (n = 2) are presented as a negative control.

To further assess the role of Cx3cr1 in hepatic insulin signaling, we injected an insulin bolus into the inferior vena cava of WT and Cx3cr1^-/-^ mice, harvested liver and adipose tissue and performed immunoblotting of insulin signaling molecules. After 20 weeks of HFD, relative to WT mice, there were significantly greater levels of phosphorylated AKT and a trend toward higher levels of serine 21 phosphorylated GSK3β following acute insulin administration into livers of Cx3cr1^-/-^ mice **(Fig 3)**. No difference was seen in adipose tissue levels of these molecules (data not shown). Levels of mRNA for insulin signaling genes in liver on chow and 4, 12 and 24 weeks high-fat diet did not differ significantly between Cx3cr1^-/-^ and wild-type mice **(Table 1)**.

**Table 1:** There was no difference in liver insulin signaling gene expression between WT and Cx3cr1^-/-^ mice on chow or high-fat diet.

GENE	Lean*		4 week HFD		12 week HFD		24 week HFD	
	WT	CX3CR1^-/-^	WT	CX3CR1^-/-^	WT	CX3CR1^-/-^	WT	CX3CR1^-/-^
GSK3α	1.0	1.2+/-0.7	0.7+/-0.2	1.2+/-0.6	1.2+/-0.8	1.3+/-0.8	1.8+/-0.7	1.8+/-0.6
IRS1	1.0	1.2+/-1.1	0.5+/-0.5	2.4+/-2.0	1.3+/-0.8	1.7+/-1.6	1.2+/-1.0	2.0+/-1.2
IGFBP-1	1.0	2.7+/-3.9	0.7+/-0.5	1.0+/-1.9	0.7+/-0.6	1.9+/-3.3	0.6+/-1.0	0.3+/-0.3
GSK3β	1.0	1.2+/-0.8	0.4+/-0.2	1.1+/-0.7	1.1+/-0.5	1.2+/-0.9	1.2+/-0.5	1.5+/-0.6
GLUT2	1.0	1.3+/-0.6	0.7+/-0.3	1.3+/-0.8	1.5+/-0.6	1.7+/-0.8	1.8+/-0.7	2.2+/-0.7
AKT	1.0	0.9+/-0.4	0.3+/-0.2	1.2+/-0.9	0.9+/-0.4	1.2+/-1.0	1.0+/-0.4	1.2+/-0.5
FOXO1	1.0	1.7+/-0.9	1.1+/-0.3	1.3+/-1.8	1.0+/-0.4	1.3+/-0.8	3.4+/-5.4	1.0+/-1.1
IRS2	1.0	1.9+/-2.4	0.9+/-0.6	1.1+/-1.7	1.0+/-0.7	1.2+/-0.8	1.2+/-1.8	0.7+/-1.2

Analysis of gene expression by qPCR showed no differences between groups on chow diet or short-, medium-, and long-term high fat feeding.

*Mean and standard deviation of expression levels relative to WT lean are shown.

N = 4/group lean and 12/group 4 week, 12 week and 24 week HFD. P = NS between WT and CX3CR1^-/-^ for all genes and time points shown

### Cx3cr1^-/-^ had no impact on adipose tissue macrophage populations on HFD, but did reduce circulating Ly6C^lo^ monocyte population

**Fig 4:**
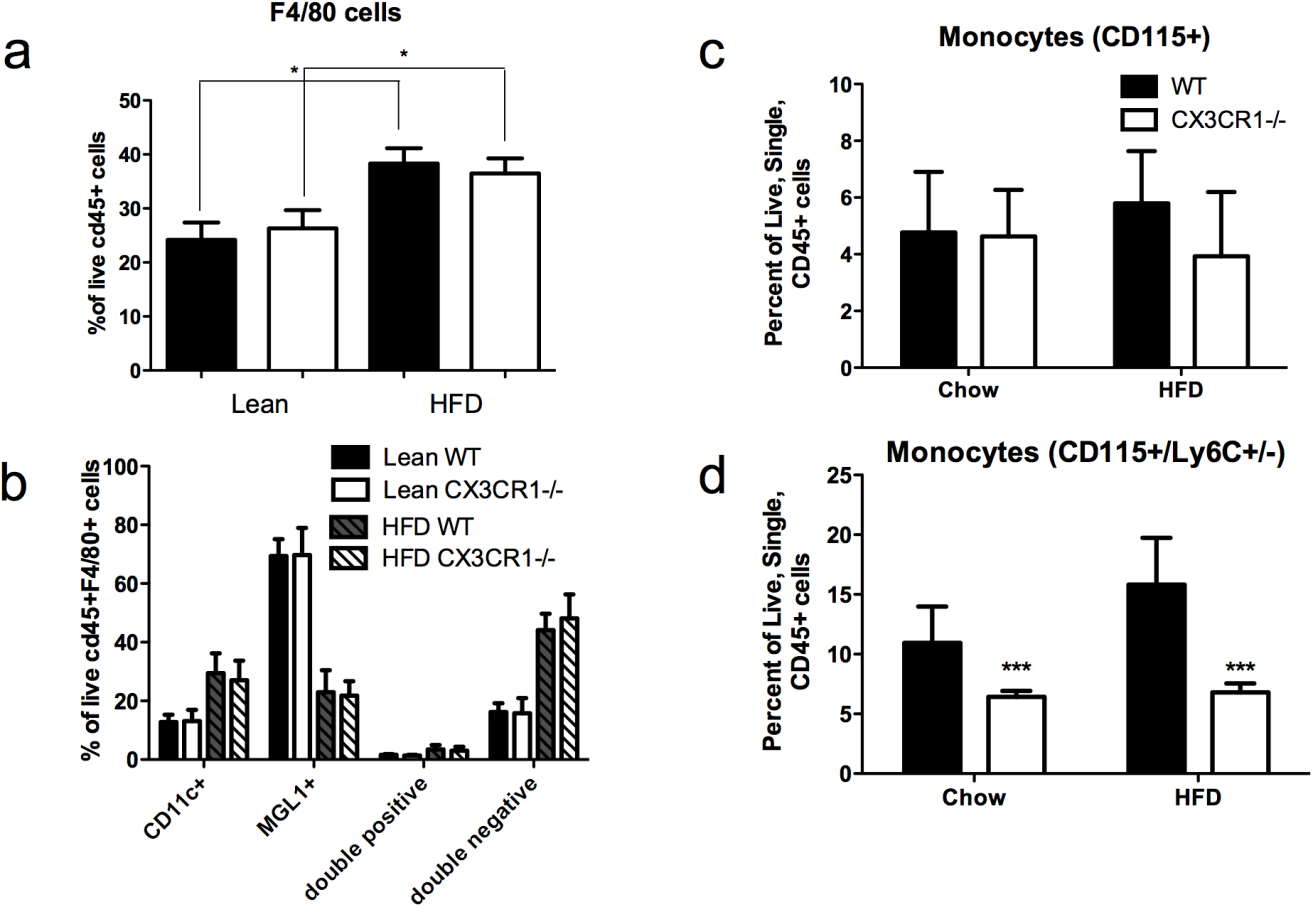
Cx3cr1^-/-^ had no impact on adipose tissue macrophage populations on high-fat diet but did cause reduction in circulating Ly6C^lo^ monocyte population. Flow cytometry of adipose tissue (n = 8/group) in lean and obese (24 week HFD) mice showed expected increases in total macrophages, cd11c+ M1 macrophages, and double negative macrophages with high-fat feeding, but no difference in a) total macrophage population or b) macrophage M1 and M2 subtypes between groups (n = 8/group). Flow cytometry of blood (n = 10/group) showed no difference in c) total monocytes though there was d) expected decrease in percentage of Ly6C^lo^ monocytes (and thus reciprocal increase in percentage of Ly6C^hi^ monocytes) in Cx3cr1^-/-^ compared to WT mice.

Although HFD increased adipose macrophages, and the proportion of M1 vs. M2 subtypes neither the total adipose tissue macrophage count nor the proportion of M1 vs. M2 subtypes was influenced by Cx3cr1 deficiency. On chow diet, total cd11b+ F4/80^hi^ macrophages (as percent of live cd45+ cells) were identical between groups (24.2 +/- 5.54 in WT vs. 26.3 +/- 5.84 in Cx3cr1-/-, p = 0.4). HFD increased the total macrophages, but this was similar in Cx3cr1^-/-^ and WT mice (38.33 +/-6.90 in WT vs. 36.47 +/- 3.87 in Cx3cr1-/-, p = 0.7) **(Fig 4A)**. Similarly, there were identical percentages of M1 (cd11c+MGL1-) macrophages (12.8 +/- 2.54 vs. 13.1 +/- 3.87; p = 0.99), M2 (cd11c-MGL1+) macrophages (69.4 +/- 5.76 vs. 69.7 +/- 9.22; p = 0.99) and double negative (cd11c-MGL1-) macrophages (16.2+/- 3.04 vs. 15.8 +/- 5.17; p = 0.70) at 2 weeks HFD; after 20-week HFD both groups had equally increased M1 (29.5+/-6.7 vs. 27.1 +/- 6.67; p = 0.81), decreased M2 (23.0 +/-7.45 vs. 21.8 +/- 4.96; p = 0.99) and increased double negative macrophages (44.1+/-5.64 vs. 48.1 +/- 8.14; p = 0.48). Double positive (cd11c+MGL1+) were also similar in both groups and did not change with HFD **(Fig 4B)** in WT and Cx3cr1^-/-^ mice respectively.

Flow cytometry of blood showed no significant difference in total monocyte population between groups (**Fig 4C**) but revealed expected[23] reduction in percentage of Ly6c^lo^ monocytes in Cx3cr1^-/-^ mice on chow and HFD (chow diet: 6.4 +/- 1.7% vs. 10.9 +/- 3.0% of live, cd45+ cells, p<0.001; 10 week high-fat diet: 6.8 +/- 2.0% vs. 15.8 +/- 3.9%, p<0.001) (**Fig 4D**). There was a reciprocal increase in percentage of Ly6C^hi^ monocyte in the Cx3cr1^-/-^ group (data not shown).

### Cx3cr1^-/-^ increases circulating Fractalkine levels without change in inflammatory or metabolic markers

**Fig 5:**
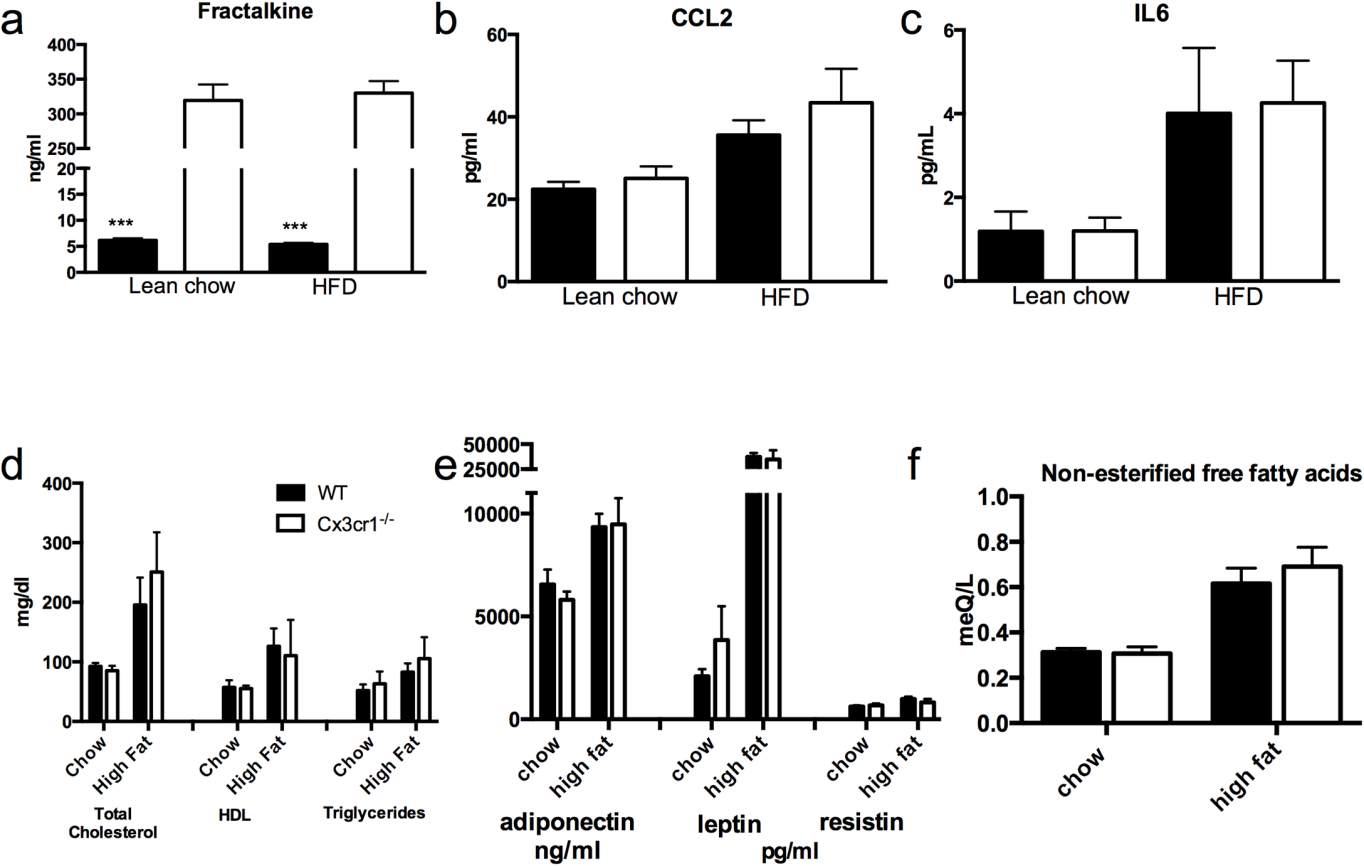
Cx3cr1^-/-^ increases circulating fractalkine levels but has no effect on other inflammatory or metabolic markers in chow or high-fat fed mice. (a) Circulating fractalkine levels by ELISA were more than 50-times higher in Cx3cr1^-/-^ mice (p<0.001) at chow and 18 week HFD. There was no difference between groups in the circulating inflammatory markers (b) CCL2 and (c) IL6 at any time point (n = 10/group for all). There was also no difference between groups in (d) total cholesterol, HDL, or triglycerides or (e) the adipokines adiponectin, leptin, and resistin (n = 8/group). Adiponectin is shown in ng/ml while leptin and resistin are in pg/ml. Fasting non-esterified fatty acids were also equivalent between groups (f). ***p<0.001 between groups.

Circulating levels of fractalkine were markedly elevated in Cx3cr1^-/-^ mice at all time points; this has not been reported previously but is consistent with physiological negative feedback of Cx3cr1 signaling on fractalkine production or clearance in the wild-type setting **(Fig 5A)**. Plasma levels of IL6 and CCL2 did not differ between Cx3cr1^-/-^ and WT mice groups before or during high-fat diet **(Fig 5B and 5C)**. Furthermore, circulating metabolic profiles, including circulating lipids (**Fig 5D**) and adipokines (adiponectin, leptin, and resistin) were not affected by Cx3cr1 deficiency **(Fig 5E).** Finally fasting non-esterified fatty acids were identical between groups in both chow and HFD state **(Fig 5F)**. Liver and adipose expression of TNFα, IL6, and IL1beta, and SOCS 3 and adipose levels of CX3CL1 and CCL2 were no different between groups on chow diet or after 4, 12, and 24 weeks of high fat diet (data not shown).

## Discussion

To investigate further the emerging role of the fractalkine pathway in diet-induced insulin resistance, glucose homeostasis and diabetes, we undertook detailed metabolic phenotyping in the Cx3cr1^-/-^ mouse model. Based on glucose tolerance testing and hyperinsulinemic clamps, our results showed a moderate and reproducible protective effect of Cx3cr1 deficiency on HFD-induced glucose intolerance, and enhanced hepatic insulin sensitivity. Unlike one report, we did not observe an impact of Cx3cr1 deficiency on insulin secretion in mice fed normal chow or HFD. As noted by others[23], we did not observe an effect of Cx3cr1 deficiency on systemic or adipose-specific inflammation or macrophage infiltration of adipose in our studies.

Work by our group and others have suggested that CX3CL1-CX3CR1 signaling may modulate glucose homeostasis and insulin resistance in humans. We reported that circulating CX3CL1 levels were higher in subjects with diabetes than without[7]. Sirois-Gagnon et al. reported an association of genetic variants in CX3CR1, the exclusive receptor for CX3CL1, with obesity in humans and we also found nominal association of CX3CR1 genetic variation with obesity and type 2 diabetes[7, 15]. A recent cohort study demonstrated that subjects with metabolic syndrome had higher serum CX3CL1 levels at baseline than those without, and that baseline CX3CL1 levels were an independent predictor of development of metabolic syndrome over a 2 year follow-up period[24]. In a study of pregnant women with and without gestational diabetes, serum CX3CL1 levels were similar between groups, but independently associated with HOMA-IR, suggesting effects on insulin signaling[25].

Although mice can provide powerful genetic tools for probing mechanistic insights in disease, such models have produced conflicting data and raised many questions with regard to the influence of the fractalkine pathway on glucose homeostasis and metabolism. By performing detailed metabolic and tissue-level analyses during chronic HFD in Cx3cr1^-/-^ and WT litter mate mice, as well as utilizing hyperinsulinemic euglycemic clamps with tracers to assess whole body and organ specific insulin sensitivity, our studies were designed to detect subtle effects of Cx3cr1 deficiency on the metabolic responses to dietary excess and obesity. Studying multiple cohorts of mice at different time points, we detected consistent modest protective effects of Cx3cr1^-/-^ on diet induced insulin resistance mediated partly through preservation of hepatic insulin sensitivity and signaling.

Our murine studies, however, present only one facet of an evolving and conflicting picture for the role of this chemokine pathway in metabolic physiologies. In Morris et al’s studies of a fractalkine deficient model (Cx3cr1 gfp/gfp compared to heterozygote Cx3cr1 +/gfp) fed a 60% high fat diet for 20 weeks, Cx3cr1^-/-^ did not attenuate adipose inflammation or the development of peripheral insulin resistance, assessed by intraperitoneal glucose tolerance test and insulin tolerance test at 20 weeks. In their model, fasting insulin values on chow diet and after 20 weeks HFD were also identical between groups[23]. However, insulin resistance was not assessed over time and the gold standard methodology of hyperinsulinemic-eugylemic clamps were not performed. Furthermore, subtle physiological differences in glucose homeostasis may have been overwhelmed after 20 weeks on a 60% HFD.

Polyak et al, using a similar mouse model (Cx3cr1 gfp/gfp compared to Cx3cr1 +/gfp), but feeding a fat-enriched diet (2:1 mixture of chow and lard) for only 10 weeks, reported that the Cx3cr1 null mice had decreased weight gain and lesser visceral white adipose tissue as well as protection from glucose intolerance and adipose inflammation[26]. In their studies, Cx3cr1 gfp/gfp mice had similar fasting glucose levels but lower glucose levels with an intraperitoneal glucose tolerance test compared to Cx3cr1 +/gfp mice on chow and high-fat diet. The group also found that the Cx3cr1 gfp/gfp mice had reduced adipose tissue F4/80+ MHCII cells, decreased adipose tissue expression of inflammatory genes, and decreased circulating inflammatory markers. The discrepancy in findings between these studies as well as with our current work may be related to differences in time course and diet composition, with the fat-enriched diet potentially being more inflammatory, and thus enhancing subtle differences in inflammatory phenotype between groups.

In direct contrast to both of the previous studies and to our findings highlighted above, Lee et al. reported that in a different genetic model of Cx3cr1 deficiency resulted in a defect in pancreatic insulin secretion[16] and, as a direct result the Cx3cr1^-/-^ mice developed glucose intolerance and overt hyperglycemia on 60% high-fat diet. In these studies, relative to WT mice, the Cx3cr1^-/-^ mice had lower fasting and glucose stimulated insulin secretion at baseline and on HFD, though weight and systemic and adipose tissue inflammatory markers were not different. Notably, the mouse genetic model predominantly employed was different to that in all other published studies examining metabolic effects of Cx3cl1/Cx3cr1 signaling, including our work. Overall, these disparate findings suggest effects specific to the genetic knockout strategies employed and that subtle metabolic and tissue inflammatory effects may be masked or revealed based on the intensity and time-course of the high-fat diet challenge.

Although our findings suggest a specific impact of Cx3cr1 on hepatic insulin signaling, this work does not yet reveal the fundamental molecular mechanisms underpinning this effect. Moreover, it is unclear as to which organs are responsible for increasing peripheral insulin sensitivity in Cx3cr1^-/-^ mice. Recently, Morari et al. described a role for the fractalkine pathway in HFD-induced hypothalamic inflammation via the toll-like receptor 4 pathway[27]. The group found that administration of a small interfering RNA against fractalkine injected into the hypothalamus also led to weight loss, compared to mice injected with scrambled RNA. While we did not see reduction in weight in our Cx3cr1^-/-^ mice to suggest such hypothalamic involvement, the possibility of fractalkine activity in this region does require further exploration, particularly in light of the growing body of evidence regarding the role of the hypothalamus in glucose homeostasis[28]. Further studies in rodent models and humans are required to probe such mechanisms and to resolve differences between published rodent models as well as to determine if physiological effects of CX3CR1 loss and gain of function are fully consistent between humans and rodent models in obesity, glucose homeostasis and diabetes. Such work may ultimately translate into therapeutic strategies targeting individual or combinations of chemokine receptors as preventive strategies or disease treatment.

In conclusion, we demonstrate a moderate and reproducible protective effect of Cx3cr1 deficiency on glucose intolerance and insulin resistance. This work provides the context for continued mechanistic studies of the CX3CL1 pathway, in concert with other chemokines, on glucometabolic as well as cardiovascular traits, specific interrogation of human chemokine genetics in these traits and the potential for development of novel therapies targeting these pathways.

## Supporting Information

S1 Checklist ARRIVE Guidelines Checklist. Animal Research: Reporting In Vivo Experiments[18].
(PDF)
